# Aerobic Vaginitis Induced by *Escherichia coli* Infection During Pregnancy Can Result in Adverse Pregnancy Outcomes Through the IL-4/JAK-1/STAT-6 Pathway

**DOI:** 10.3389/fmicb.2021.651426

**Published:** 2021-04-07

**Authors:** Chong Fan, Youjin Dai, Lei Zhang, Can Rui, Xinyan Wang, Ting Luan, Yuru Fan, Zhiyong Dong, Wenwen Hou, Ping Li, Qinping Liao, Xin Zeng

**Affiliations:** ^1^Women’s Hospital of Nanjing Medical University, Nanjing Maternity and Child Health Care Hospital, Nanjing, China; ^2^Key Laboratory of Model Animal Research, Animal Core Facility of Nanjing Medical University, Nanjing Medical University, Nanjing, China; ^3^Department of Obstetrics and Gynecology, Beijing Tsinghua Changgung Hospital, School of Clinical Medicine, Tsinghua University, Beijing, China

**Keywords:** aerobic vaginitis, *Escherichia coli*, IL-4/JAK-1/STAT-6 pathway, decidual macrophages, vaginal microbiome

## Abstract

Aerobic vaginitis (AV) can occur if normal vaginal microflora are dominated by aerobic bacteria, seriously affects not only female health, but also fetal health while they are pregnant. Besides, pregnant status also aggravates the symptoms and consequences of the infection. Here, we infected pregnant BALB/c mice with *Escherichia coli* on embryonic day 4.5 (E4.5) (study group), and administered an equivalent volume of phosphate-buffered saline in another cohort of pregnant mice (control group). We recorded the weight of pregnant mice and their fetuses. The maternal and fetal weight of the study group decreased in comparison with that of the control group, whereas the weight of placenta increased in the study group. Then, five genes with significant upregulation and 15 genes with downregulation were screened. Expression of interleukin 4 (IL-4) mRNA in the study group decreased to 18.5%. Enzyme-linked immunosorbent assay results showed IL-4 expression in mouse plasma declined in the study group at E11.5 and E18.5. mRNA expression of chemokine (c-c motif) ligand (CCL)-17, CCL-22, CCL-24, IL-4, Janus Kinase (JAK)-1, signal transducer and activator of transcription (STAT)-6, and GATA-3 showed significant downregulation in placental and uterine tissues. Flow cytometry of primary decidual macrophages (DMs) revealed more M1-like macrophages in the study group. And after addition of IL-4 to DMs, more M1 macrophages polarized to M2 type macrophages. We did not discover bacteria existed in mouse placentas. Our study affords a feasible method for exploring and managing AV during pregnancy.

## Introduction

The human microbiome has attracted considerable attention in the past few years (Lloyd-Price et al., 2016). As a vital compartment of the human microbiome, the vaginal microbiome, usually predominated by conspecific *Lactobacillus*, is increasingly well recognized by scientific researchers (Fettweis et al., 2019). If vaginal lactobacilli are absent but polymicrobial flora (e.g., bacteria of genera *Gardnerella*, *Atopobium*, *Prevotella*, *Peptostreptococcus*, *Mobiluncus*, *Sneathia*, *Leptotrichia*, or *Mycoplasma*) are present, bacterial vaginosis occurs (Onderdonk et al., 2016). If the prevailing organisms are disrupted and governed by aerobic bacteria, such as *Escherichia coli* (*E. coli*), group B *Streptococci*, or *Staphylococcus aureus*, a disturbing state called “aerobic vaginitis” (AV) emerges (Donders et al., 2002, 2011, 2017). The clinical complaints of patients with AV usually include viscous and yellow vaginal discharge, stinging, burning sensations, and even dyspareunia (Donders et al., 2017). Therefore, AV merits increased attention.

Infection, including AV, usually denotes reactions in local tissue and a systemic inflammatory reaction caused by invasion of bacteria, fungi, viruses, parasites, or other pathogens. The immune reactions induced by infections may have a negative impact on the pregnancy outcome, such as an intrauterine infection, abortion, preterm birth, or an ectopic pregnancy (Smith and Ravel, 2017). Moreover, an increased risk of infection occurs during pregnancy (Sacerdoti et al., 2018). Gabriel and Arck (2014) indicated that, compared with non-pregnant women, pregnant women were seven-times more likely to be hospitalized due to influenza, and two-times more likely to die. These phenomena may be the result of changing hormone levels. A high level of estrogen during pregnancy has an anti-inflammatory action but makes T cells skew toward T-helper (Th)1 cells, which is harmful to pregnancy (Gabriel and Arck, 2014). This complicated relationship makes infection during pregnancy more intractable.

The exact pathogenesis of AV is still not known, and studies have focused mainly on local immunological changes. A previous article reports that levels of interleukin (IL)-1β, IL-6, and IL-8 were significantly increased in vaginal rinsed samples from AV patients (Marconi et al., 2013). Other studies link the inflammatory factors mentioned above with preterm delivery, neonatal sepsis, and other adverse pregnancy outcomes (Dörtbudak et al., 2005; Simonsen et al., 2014; Gomez-Lopez et al., 2019).

Not knowing the exact pathogenesis of AV hampers exploration of efficacious therapeutic methods for both pregnant and non-pregnant patients by obstetricians. In addition, the recurrence of AV and non-efficacious medications aggravate the difficulty of AV treatment (Donders et al., 2011; Han et al., 2019). Moreover, the drugs used during pregnancy are controversial (Bailey et al., 2018; Halpern et al., 2019). All of these factors elicit numerous difficulties for patients and obstetricians. Therefore, an in-depth study is imperative to alleviating the discomfort of AV patients and to improving the current situation of pregnant women who suffer from AV.

In the current study, a mouse model of AV base *E. coli* infection during pregnancy was created, and the effects on the mother and fetus were observed. Subsequently, the underlying mechanisms of action of the vaginal infections induced by *E. coli* infection on pregnancy outcome were investigated. We also explored whether vaginal infections ascended to the placenta.

## Materials and Methods

### Preparation of *Escherichia coli*

*Escherichia coli* strain CTL5034 was derived from the Microbiological Laboratory of Nanjing Drum Tower Hospital (Nanjing, China). It was cryopreserved in 15% glycerinum at −80°C. The detailed methods of cultivating and preserving *E. coli* followed those outlined in our previous work (Jiang et al., 2019). For preparation of *E. coli* suspensions, *E. coli* was melted softly on ice, and then mixed with configured Luria-Bertani (LB) medium at a ratio of 1 to 50, into a tube that was agitated slowly. Subsequently, the tube was placed in a constant temperature incubator with a shaker at 220 revolutions per minute (rpm) for 12 to 16 h.

### Animal Experiments

All animal procedures were approved (IACUC-2008043) by the Animal Research Ethics Board of Nanjing Medical University (Nanjing, China). All male and female BALB/c mice (6–8 weeks) were purchased from Beijing Vital River Laboratory Animal Technology (Beijing, China). They were raised in the specific pathogen free (SPF) laboratory environment in the Animal Care Facility of Nanjing Medical University.

A male mouse cohabited with two female mice in the afternoon. The vaginal plug was checked the next day, and pregnant mice were recorded as “embryonic day 0.5” (E0.5). Female mice were then classified randomly into the study group and the control group.

We infused 20 μL of *E. coli* culture suspension (the dose was about 2 × 10^7^ CFU) and an equivalent phosphate-buffered saline (PBS) into the vagina at E4.5 in the study group and the control group, respectively. The mice were placed in a comfortable position for *E. coli* or PBS administration into the vagina. After inoculation, the mice were held still in the same position for more than 3 min. We then douched the mice vagina with a total of 30 μL PBS (3 × 10 μL) at different time points. Next, we gathered the vaginal lavage fluid (VLF) in aseptic Eppendorf (EP) tubes at E8.5, E11.5, and E18.5. We also carried out cesarean section and collected the required tissues at E8.5, E11.5, and E18.5 under sterile conditions. At the same time as the cesarean section, the weight of fetuses and the placenta was recorded at E18.5. The weight of mice at E0.5, E4.5, E8.5, E11.5, and E18.5 was also documented.

### Vaginal Lavage Fluid Culture

Vaginal lavage fluid was diluted in a series of concentrations with sterile PBS around an alcohol lamp on a clean bench. Subsequently, 10 μL of diluted VLF was coated uniformly upon a prepared solid LB plate medium by Bioclean coated glass rod. Afterward, the plate medium was placed in a constant temperature cabinet at 37°C.

### Gram Staining

The procedures of Gram staining involved the preparation and staining of smears, including primary staining, mordanting, decolorizing, and counterstaining. First, an inoculation loop was sterilized in the flame of an alcohol lamp until red-hot, and then allowed to cool naturally for ∼30 s. Next, we picked one suitable colony using the inoculation loop onto the slide and coated it evenly, followed by passing the slide softly but quickly through the flame three times to fix the smears. After smear fixation, we placed one drop Crystal Violet solution onto the smear for ∼1 min to achieve primary staining. For mordanting, Gram’s iodine solution was covered onto the smear for ∼1 min. Then, 95% ethanol was added to the slide to decolorize the stain for at most 30 s. Last, we covered the slide with safranin for 1 min. After washing and air drying, we observed the staining results under a microscope (Carl Zeiss, Jena, Germany). Purple represented Gram-positive bacteria and pink denoted Gram-negative bacteria.

### Hematoxylin-Eosin Staining (H&E)

Before staining, paraffin-embedded tissue sections were created. Briefly, fresh vaginal tissue was fixed with 4% paraformaldehyde solution for over 24 h. The tissue was then trimmed with a scalpel. Subsequently, dehydration and diaphanization were carried out in an automatic tissue processor. Next, tissue was embedded with paraffin and sections were created. The prepared paraffin sections with a thickness of 4 μm were then dewaxed with xylene and gradient concentrations of ethanol. After dewaxing and washing, the sections were stained with hematoxylin (3–8 min) and eosin (1–3 min) in turn. Sections were placed in different concentrations of ethanol and xylene for dehydration. After air drying, we mounted the sections in synthetic resin and then observed the dyeing results *via* a microscope. The nucleus displayed as blue, while the cytoplasm displayed as red.

### Polymerase Chain Reaction (PCR) Array

The Mouse Cytokines and Chemokines RT^2^ Profiler PCR Array (QIAGEN, Düsseldorf, Germany) was used in this trial. According to the manufacturer’s instructions, we transferred 10 μL of the reaction mixture to each well of a 384-well reaction plate. Subsequent reaction conditions were as follows: a cycle of heating for 10 min at 95°C, 40 cycles of collecting fluorescence data for 15 s at 95°C, and 30 s at 60°C.

### Separation and Culture of Cells

Samples of the uterus and placenta tissue were obtained by cesarean section at E11.5 and washed to clear with PBS immediately. A 10 mM 4-(2-hydroxyethyl) piperazin-1-ethanesulfonic aci (HEPES), 1% collagenase type IV, and 0.5% bovine serum albumin were then dissolved in RPMI – 1,640 medium containing 10% fetal bovine serum (FBS) followed by a detachment solution. Subsequently, the tissue was cut into pieces using the prepared detachment solution and these pieces were placed in a centrifuge tube. A 10 to 15 mL of the detachment solution was then added, and the tube was placed on an incubator shaker at 150 rpm for 30 min at 37°C. Next, the cell suspension was transferred to a new centrifuge tube, and the precipitate was detached again with 10 mL of the detachment solution on the incubator shaker at 150 rpm for 20 min at 37°C. The cell suspension was then collected into the old centrifuge tube, allowed to stand for ∼15 min on ice, and then centrifuged at 2,000 rpm for 10 min at 4°C. The supernatant was removed, and the precipitate was centrifuged again after resuspension with PBS. The precipitate was then resuspended with 3 mL of 40% percoll, and the suspended liquid was added very slowly to 2 mL of 70% percoll, followed by gradient centrifugation at 2,500 rpm for 10 min at 20°C. Afterward, the buffy coat in the middle was transferred to a new centrifuge tube and filled with sterile PBS. After centrifugation at 2,000 rpm for 10 min at 4°C, the supernatant was discarded, and the cell pellet was resuspended with 1 mL of PBS. Red Blood Cell Lysis Buffer was applied when red blood cells could be clearly observed. Finally, the separated cells were divided into two parts and IL-4 was added to one of the parts. Both parts were cultured with RPMI – 1,640 medium containing 10% FBS at 37°C in a humidified incubator in an atmosphere of 5% CO_2_.

### Flow Cytometry

Separated decidual macrophages (DMs) from mice were stimulated for 5 h with phorbol 12-myristate 13-acetate (PMA), ionomycin (Ion), and Brefeldin A (BFA). Next, DMs were collected in EP tubes and centrifuged at 1,000 rpm for 5 min at 4°C. The supernatant was then discarded and the cell pellet was resuspended using PBS. After another centrifugation, 100 μL of the liquid was retained and sealed with 5 μL of serum for 30 min in a dark environment at 4°C. Subsequently, 1 μL of antibody (0.2 mg/mL) was added to the cell suspension and incubation was allowed in the dark for 30 min at 4°C. Finally, a flow cytometry analysis was carried out on BD FACSAria^TM^ Special Order Research Product (BD Bioscience, San Jose, CA, United States).

### Isolation of Total RNA and Real-Time Reverse Transcription-Quantitative Polymerase Chain Reaction (RT-qPCR)

RNA isolation was conducted on ice. Samples of uterus or placenta tissue were lysed by the addition of the appropriate amount (1 mL of TRIzol^®^ reagent for at most 100 mg tissue) of TRIzol^®^ Reagent (Invitrogen, Carlsbad, CA, United States) for 10 min. After addition of one-fifth volume of chloroform to the homogenate, the sample was mixed fiercely for 10 s and allowed to stand for 5 min at room temperature. After centrifugation, the upper layer (transparent) was collected, and half the volume of isopropanol was added. After another round of centrifugation, the supernatant was removed carefully, and 1 mL of 75% ethanol was added to the white precipitation. After yet another round of centrifugation, the supernatant was discarded. After air-drying in a fume cupboard, 40 μL of diethylpyrocarbonate was added to dissolve the extraction. Finally, the concentration of extracted RNA was measured.

For RNA isolation of extracted decidual macrophages, after rinsing with PBS for two times, 0.5 mL of TRIzol^®^ Reagent was added to each well of 12-well plates. Followed by blowing the cells and adding one-fifth volume of chloroform, the subsequent steps were the same as RNA isolation from samples of the uterus or placenta tissue.

RT-qPCR was achieved using PrimeScript^TM^ RT Reagent Kit with gDNA Eraser (TaKaRa Bio Inc., Tokyo, Japan), SYBR^®^ *Premix Ex Taq* II (Tli RNaseH Plus) (TaKaRa Bio Inc., Tokyo, Japan), and the ViiA^TM^ 7 Real-Time PCR System (Thermo Fisher Scientific, Waltham, MA, United States). Primer sets are shown as Supplementary Table 1.

### Enzyme Linked Immunosorbent Assay (ELISA)

First, we added prepared standards (100 μL) and samples (100 μL) per well into a microtiter plate, and then sealed the plate and allowed incubation for 90 min at 37°C. Subsequently, we washed the plate four times with wash buffer and added a working solution of biotinylated antibody (100 μL/well). After sealing the plate again and allowing incubation for 60 min at 37°C, the free ingredients were removed by washing four times with wash buffer. Then, 100 μL of enzyme binding working fluid was added to each well. The plate was sealed, and incubation was allowed for 30 min at 37°C. Afterward, unbound compounds were rinsed four times as mentioned before and 100 μL chromogenic agent was added to each well. After Incubation in the dark for 10 to 20 min at 37°C, a stopping solution (100 μL/well) was added. After mixing uniformly, the absorbance was measured at 450 nm immediately.

### Isolation of Microbial Genomic DNA From Placenta Tissue and PCR

Extraction of microbial genomic DNA from placenta tissue was done by the QIAamp^®^ PowerFecal^®^ Pro DNA Kit (QIAGEN, Düsseldorf, Germany). Afterward, PCR was conducted as described below. Briefly, 0.1 μg of template DNA, 0.8 μL of reverse primer, 0.8 μL of forward primer, and 10 μL of Taq Master Mix (Vazyme Biotech Co., Ltd., Nanjing, China) were added to a clean tube according to the manufacturer’s instructions. Next, we added double distilled water to obtain a reaction mixture total of 20 μL. The mixture was then PCR processed for 35 cycles as follows: denaturation for 15 s at 95°C, annealing for 15 s at 58°C, and extension for 60 s at 72°C. Bacterial 16S rRNA primers are recorded in Supplementary Table 1.

### Agarose Gel Electrophoresis

One percent gels were prepared by adding 0.5 g agarose and 50 mL of 1 × TAE buffer. Before cooling, 2.5 μL of Gold View^TM^ Staining Dye was added. After gel preparation, 5 μL of sample and 1 μL of 6 × loading buffer, as well as 6 μL of DNA marker were carefully added to each hole. Electrophoresis was subsequently performed, and the electrophoretic band was observed on the FluorChem^TM^ System (ProteinSimple, San Jose, CA, United States).

### Statistical Analysis

As for continuous data, a normality test was evaluated using the Shapiro-Wilk test. The Student’s *t*-test was used for data with a normal distribution. The Mann-Whitney *U*-test was employed for data with a non-normal distribution. All results were presented as means ± standard deviation (SD). Analyses of PCR Array was implemented using the Internet website provided by QIAGEN^1^. Expression of target genes was calculated using the 2^−ΔΔCT^ method. GraphPad Prism 8.0.1 software (GraphPad Software, San Diego, CA, United States) and SPSS 25.0 (SPSS Inc., Chicago, IL, United States) were applied for data visualization and statistical analyses. In all comparisons, a *P* value < 0.05 was considered as statistically significant.

## Results

### Establishment of a Model of Vaginal Infection With *E. coli* in Mice

**FIGURE 1:**
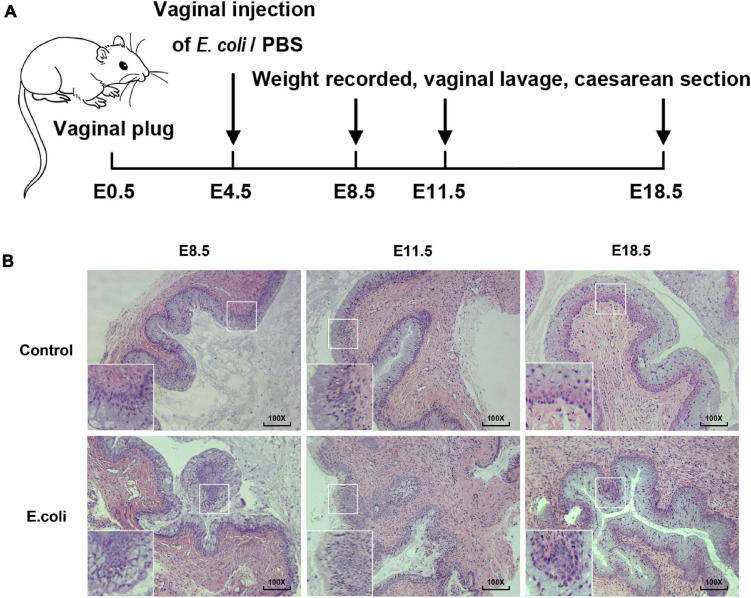
Construction of animal models of vaginal infection induced by *E. coli*. **(A)** The flow of animal experiments in this study. **(B)** H&E staining of vaginal tissue in the study group and the control group of mice at E8.5, E11.5, and E18.5. The magnification of the microscope examination used was 100×.

After examination of vaginal plugs, we infused a bacterial solution or PBS into mouse vaginas at E4.5 in the study group and control group (Figure 1A). Subsequently, VLF was acquired and cultured at E8.5, E11.5, and E18.5 for the next series of explorations and the results are displayed in Supplementary Figure 1A. Bacterial colonies were observed on the LB culture medium in the *E. coli* group. The number of bacterial colonies peaked at E8.5. The difference in the number of bacterial colonies between E11.5 and E18.5 was small, however, no bacterial clumps could be found in the control group.

Gram staining was carried out to view the morphology of bacteria and the results demonstrated that the cultured bacteria were Gram-negative bacilli (Supplementary Figure 1B), in concordant with the feature of the *E. coli*. Next, H&E staining of vaginal tissue was completed for the purpose of understanding the potential histologic changes. The magnification of the microscope examination that we used in this study was 100 × (Figure 1B). In the vaginal infection group, the infiltration of inflammatory cells in the vaginal epithelium were visible, as was the formation of lymphoid follicles. Moreover, the number of layers of the vaginal epithelium increased and the vaginal epithelium was more irregular in the study group. After H&E staining, for the *E. coli* group, changes in vaginal inflammation were not obvious as time went on. All of these results illustrate that mice exposed to *E. coli* suffered vaginal infection.

### Effects of Vaginal Infection With *E. coli* on Phenotypes of Mice and Their Offspring

Images of the fetus and placentas were obtained (Supplementary Figure 2). Images suggested that embryonic growth of mice was not yet clear at E8.5 but the fetuses looked larger than fetuses in the control group. At E11.5, the development of the extremities of the fetuses became increasingly obvious, and abortions also occurred at this time. Then at E18.5, the fetuses looked fully developed externally and larger than those in the control group. Moreover, miscarriages in the maternal mouse were distinctly visible in the *E. coli* group.

**FIGURE 2:**
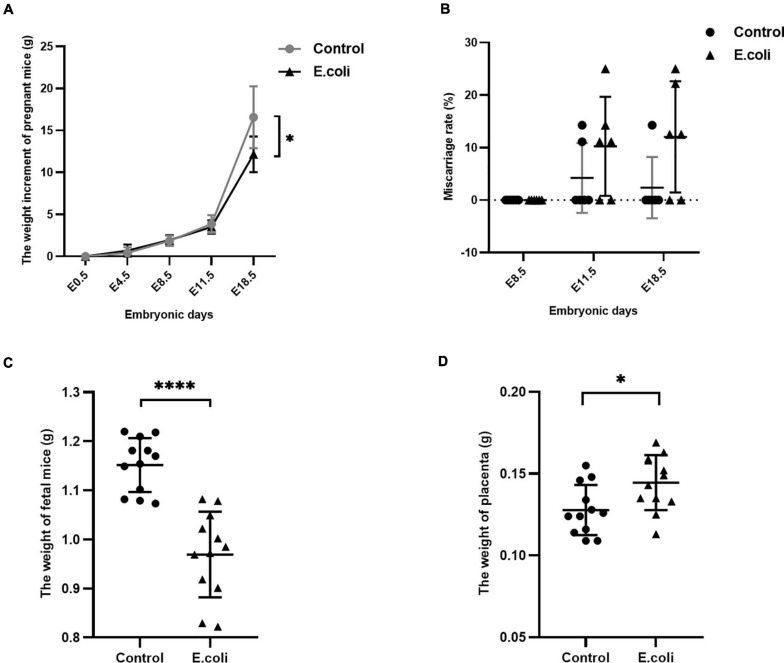
Characteristics of the mother and fetus in mice in the study (*E. coli*) group and the control group. **(A)** Weight gain of pregnant mice in the study group and control group at E0.5, E4.5, E8.5, E11.5, and E18.5. The number of mice used for calculation of weight gain of pregnant mice in the control group at E4.5, E8.5, E11.5, and E18.5 were 17, 17, 11, and 6, respectively. The number of mice used for calculation of weight gain of pregnant mice in the study group at E4.5, E8.5, E11.5, and E18.5 were 18, 18, 12, and 6, respectively. **(B)** Miscarriage rate in the study group and control group at E8.5, E11.5, and E18.5. The number of mice used for calculation of miscarriage rate in the control group at E8.5, E11.5, and E18.5 are 6, 5, and 6, respectively. The number of mice used for calculation of miscarriage rate in the study group at E8.5, E11.5, and E18.5 are 6, 6, and 6, respectively. **(C)** Weight of fetus in the study group and control group at cesarean section (*n* = 12). **(D)** Weight of the placenta in the study group and control group at cesarean section (*n* = 12). Data are presented as mean ± standard deviation. Unpaired *t*-test was used for two-group comparisons of the weight gain, the weight of fetus, and the weight of the placenta. Mann-Whitney *U*-test was used for two-group comparisons of the miscarriage rate. ^∗^*P* < 0.05, ^****^P < 0.0001.

The characteristics of pregnant mice and their fetuses are shown as Figure 2. We used 17 pregnant mice in the control group and 18 pregnant mice in the *E. coli* group. Cesarean sections were performed at all time points, and the number of mice used at each time point in the control group at E8.5, E11.5, and E18.5 were six, five, and six, respectively. Similarly, the number of mice used at each time point in the *E. coli* group at E8.5, E11.5, and E18.5 were six, six, and six, respectively. The weight of pregnant mice at E0.5 was set as 0 and Figure 2A indicates that the weight of pregnant mice increased with the number of embryonic days. The mean increased weight of pregnant mice at E4.5, E8.5, E11.5, and E18.5 in the control group was 0.44 ± 0.65 g, 1.86 ± 0.61 g, 3.87 ± 1.06 g, and 16.57 ± 3.70 g, respectively. The corresponding weight value in the *E. coli* group was 0.69 ± 0.73 g, 1.94 ± 0.59 g, 3.52 ± 0.79 g, and 12.15 ± 2.13 g, respectively. Subsequent data statistics illustrated that the significant difference in the weight of pregnant mice was noted at E18.5 between the control group and *E. coli* group (*P* = 0.030). The miscarriage rate of pregnant mice is shown in Figure 2B. The details of fetus survival of each pregnant mouse in the control group at E11.5 is as follows: 10 live and 0 dead fetuses, six live and one dead fetus, eight live and 0 dead fetuses, six live and 0 dead fetuses, and eight live and one dead fetus. Similarly, the details of fetus survival of each pregnant mouse in the *E. coli* group at E11.5 is as follows: eight live and one dead fetus, six live and two dead fetuses, eight live and 0 dead fetuses, nine live and 0 dead fetuses, eight live and one dead fetus, and six live and one dead fetus. The details of fetus survival of each pregnant mouse in the control group at E18.5 is as follows: 11 live and 0 dead fetuses, 10 live and 0 dead fetuses, nine live and 0 dead fetuses, eight live and 0 dead fetuses, seven live and one dead fetus, and six live and 0 dead fetuses. The details of fetus survival of each pregnant mouse in the *E. coli* group at E18.5 is as follows: six live and two dead fetuses, seven live and one dead fetus, seven live and one dead fetus, three live and 0 dead fetuses, seven live and 0 dead fetuses, and seven live and two dead fetuses. No abortion was found in either groups at E8.5, but abortions occurred from E11.5. The mean miscarriage rate in the control group was 4.23% at E11.5 and 2.38% at E18.5, simultaneously, 10.25% at E11.5, and 12.04% at E18.5 in the *E. coli* group. Though there was no statistically significant difference in the miscarriage rate, phenomena of abortion could be observed with the naked eye in the *E. coli* group. Moreover, the mean weight of fetuses (Figure 2C) at E18.5 in the control group (1.15 ± 0.06 g) was higher than that in the *E. coli* group (0.97 ± 0.09 g, *P* < 0.0001). On the contrary, the weight of the placenta (Figure 2D) was lower in the control group (0.13 ± 0.02 g) than that in the *E. coli* group (0.14 ± 0.02 g, *P* = 0.018). These consequences suggested that vaginal infection with *E. coli* led to adverse pregnancy outcomes.

Whether vaginal infections ascend to the placenta is still a controversial issue. We therefore used gel electrophoresis to explore this disputed issue (Supplementary Figure 3). To exclude contingency, three pairs of primers, that were contraposed with bacterial 16S rRNA, were designed. Moreover, for each pair of primers, four samples of genomic DNA, extracted from placentas in both groups, were used. Amplicons of bacterial 16S rRNA gene sequences were not observed by gel electrophoresis and this signified that no bacteria was found in the placenta.

### Pathway Mechanism of Screening and Preliminary Validation of Vaginal Infection With *E. coli* in Mice

**FIGURE 3:**
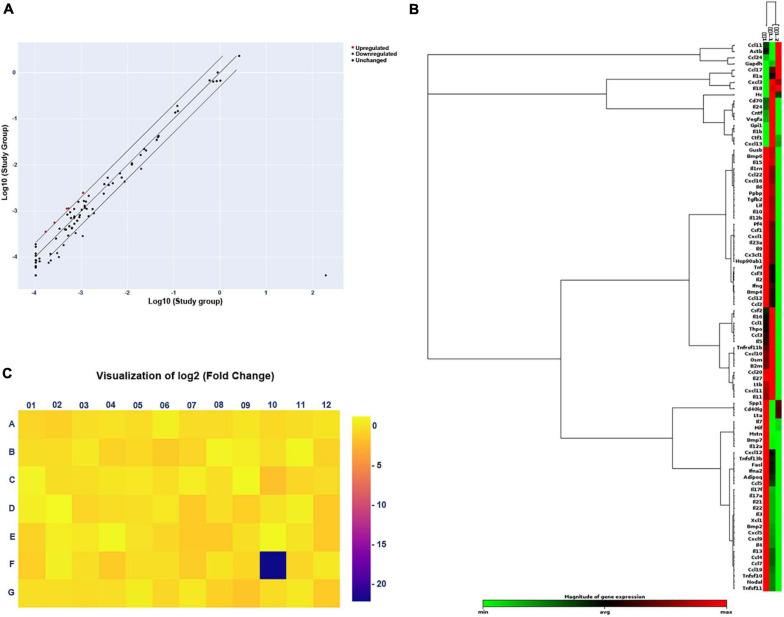
Changes in gene expression in the study (*E. coli*) group compared with that in the control group by PCR Array. **(A)** Scatter plot of the results of every gene expression. The central diagonal line indicates unchanged gene expression, while the outer diagonal lines indicate the selected fold regulation threshold. Red dots denote genes with upregulated expression, while blue dots represent genes with downregulated expression. Black dots denote genes with unchanged expression. **(B)** Clustergram of genes with significantly upregulated or downregulated expression. Each column expresses a group, and each row represents a gene. Various colors display the magnitude of gene expression and the degree of gene expression from minimum to maximum is exhibited from green to red. **(C)** Heat map of the results of every gene expression. Different colors show the different levels of gene expression. Colors ranging from blue to yellow means downregulated to upregulated gene expression.

After gel electrophoresis, we identified that ascending infections were not present. Next, PCR Array was designed for further exploration of the mechanism of adverse pregnancy outcomes. For adverse pregnancy outcomes that occurred at E11.5 (which represents the late stage of limb development), we chose the tissue at E11.5 for subsequent exploration of the mechanism of action. A scatter plot was employed to compare every gene on the PCR Array between the study and the control group (Figure 3A) and a clustergram (Figure 3B), as well as a heat map (Figure 3C) aided in visualization. Finally, five significantly upregulated genes and 15 downregulated genes were screened and the detailed significantly expressed genes, defined as the expression of the gene up- or downregulated over 2 folds, are displayed in Supplementary Table 2.

**FIGURE 4:**
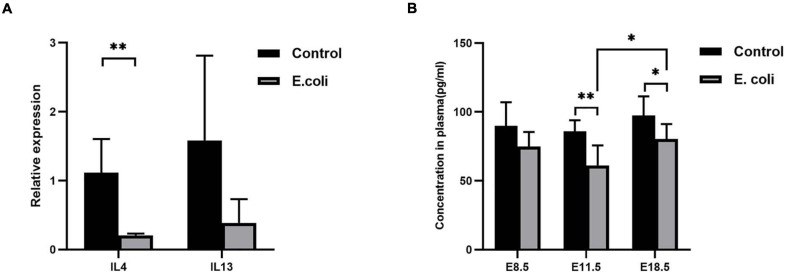
Validation of screening results of differentially expressed genes. **(A)** Validation of screened differentially expressed genes by PCR. Four samples of each group were used. The experiment was performed in triplicate and repeated two times. **(B)** The concentration of IL-4 expression in the blood of mice in the study (*E. coli*) group at E8.5, E11.5, and E18.5 (*n* = 6). Data are shown as mean ± standard deviation. Unpaired *t*-test was used for two-group comparisons. ^∗^*P* < 0.05, ^∗∗^*P* < 0.01.

From the reports of PCR Array, meanwhile, coupled with overall and deep literature consulting, interleukin (IL)-4 and IL-13 were chosen for further research. We then used RT-qPCR for primary validation of IL-4 and IL-13 (Figure 4A). From the results, IL-4 mRNA expression in the *E. coli* group, compared to the control group, reduced significantly to 18.5%, however, IL-13 mRNA expression showed no significant difference between the two groups.

For further confirmation of IL-4 expression, we performed ELISA using mouse plasma. ELISA data (Figure 4B) revealed that there was no significant difference in the concentration of IL-4 in plasma at E8.5, but a significantly reduced concentration of IL-4 in plasma were detected in mice in the study group compared with that in mice of the control group at E11.5 (61.13 ± 14.61 and 85.79 ± 8.17, respectively) and E18.5 (80.41 ± 10.74 and 97.46 ± 13.86, respectively). Furthermore, the level of IL-4 increased significantly at E18.5 compared with that at E11.5 in the study group.

### Expression of the IL-4/JAK-1/STAT-6 Pathway in Mice Infected With *E. coli*

**FIGURE 5:**
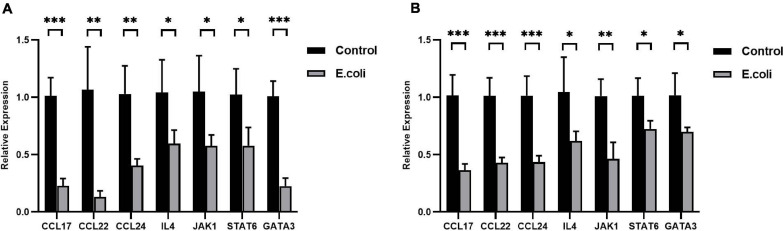
Expression of the IL-4/JAK-1/STAT-6 pathway in mice with aerobic vaginitis induced by *E. coli* infection. **(A)** Relative expression of the IL-4/JAK-1/STAT-6 pathway in the placenta of mice infected by *E. coli* at E11.5. **(B)** Relative expression of the IL-4/JAK-1/STAT-6 pathway in the uterus of mice infected by *E. coli* at E11.5. Four samples of each group were used. The experiment was performed in triplicate wells and repeated three times. Data are shown as mean ± standard deviation. Unpaired *t*-test was used for two-group comparisons. ^∗^*P* < 0.05, ^∗∗^*P* < 0.01, ^∗∗∗^*P* < 0.001.

After primary validation of the results of PCR Array, IL-4 was supposed to play a key role in vaginal infection by *E. coli*. Real-time RT-qPCR was carried out to look for potential mechanisms, which should be responsible for this phenomenon. Chemokine (c-c motif) ligand (CCL)-17, CCL-22, CCL-24, IL-4, Janus Kinase (JAK)-1, signal transducer and activator of transcription- (STAT)-6, and GATA-3 mRNA expression all exhibited significant downregulation in the placenta tissues of the study group compared with that of the control group at E11.5 (Figure 5A) and the fold change in the *E. coli* group dropped to 22.4, 12.2, 39.1, 57.2, 54.9, 56.2, and 22.4%, respectively. At E11.5, in uterus tissues, the trends in the significant gene expression were consistent with those in the placenta (Figure 5B) and the fold change in the study group reduced to 35.9, 42.3, 42.8, 59.3, 45.8, 71.6, and 68.5%, respectively.

### Decidual Macrophages Converted More to M1 Macrophages in Mice Infected With *E. coli* and This Transformation Could Be Reversed by Addition of IL-4

IL-4 plays a protective role, relating to improved vascular function and reducing inflammatory factors in successful pregnancies (Chatterjee et al., 2014; Cottrell et al., 2019). Recently, IL-4 gradually connects with DMs, which are the second largest type of leukocyte population in the maternal-fetal interface and also the major subtypes of antigen presenting cells during pregnancy (Erlebacher, 2013; Tang et al., 2015).

**FIGURE 6:**
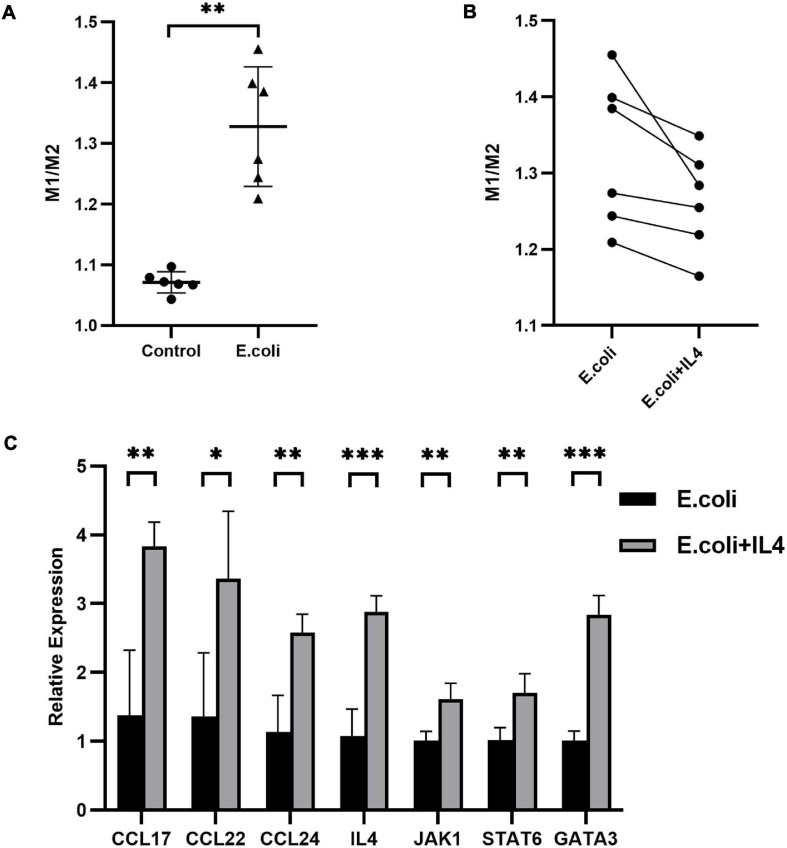
Primary decidual macrophages extracted from the maternal-fetal interface in mice. **(A)** Ratio of M1 to M2 macrophages extracted from the maternal-fetal interface between the study (*E. coli*) group and control group (*n* = 6). **(B)** Ratio of M1 to M2 macrophages before and after addition of IL-4 to macrophages extracted from the maternal-fetal interface in the study group (*n* = 6). **(C)** Relative expression of the IL-4/JAK-1/STAT-6 pathway in primary decidual macrophages extracted from the maternal-fetal interface at E11.5. Four samples of each group were used. The experiment was performed in triplicate wells and repeated two times. Data are shown as mean ± standard deviation. Unpaired *t*-test was used for two-group comparisons. ^∗^*P* < 0.05, ^∗∗^*P* < 0.01, ^∗∗∗^*P* < 0.001.

To further investigate the role of IL-4 during pregnancy in mice with vaginal infection, we isolated the DMs from the maternal-fetal interface and added IL-4 to isolated DMs of the *E. coli* group. We calculated the ratio of M1 to M2 macrophages: the average ratio in the control group and *E. coli* group was 1.071 ± 0.018 and 1.328 ± 0.099, respectively (Figure 6A). These results indicated that more M1 macrophages were found in the *E. coli* group. After addition of IL-4 to the petri dish of the *E. coli* group, all extracted cells showed decreased M1 macrophages and increased M2 macrophages (Figure 6B). In other words, M1 macrophages converted to M2 macrophages gradually after IL-4 treatment.

Next, we performed RNA extraction from isolated primary DMs to explore the alteration of mRNA expression after administration of IL-4. Compared with the *E. coli* group which was not manipulated with IL-4, mRNA expression of CCL-17, CCL-22, CCL-24, IL-4, JAK-1, STAT-6, and GATA-3 in the treatment group increased to 278, 247, 227, 268, 160, 167, and 281%, respectively (Figure 6C).

## Discussion

Infections, always a source of anxiety, that occur during pregnancy increase the risk of intrauterine growth restriction, preterm labor, chorioamnionitis, and even miscarriage or stillbirth (Giakoumelou et al., 2016; Racicot and Mor, 2017; Vornhagen et al., 2017). Pregnant women are more susceptible to infections than non-pregnant women (Sacerdoti et al., 2018; Vojtek et al., 2018). Moreover, the adverse effects of an infection can be further aggravated by pregnancy. Among these infections, vaginal infection is distinct as the vagina acts as a cavity for delivery, and ascending infections can occur. Since Donders and colleagues put forward a new state called AV in 2002, AV has entered the public consciousness and has gradually been studied thoroughly (Donders et al., 2002). In some studies, *E. coli* is listed as a common pathogen in a great many different pathogenic bacteria (Tempera et al., 2004, 2006). A previous study indicated that *E. coli* infection could result in adverse maternal and fetal complications, including intrauterine growth restriction, neonatal hemolytic uremic syndrome, spontaneous abortion, and even fetal death (Sacerdoti et al., 2018). Burdet and colleagues reported that the generation of prostaglandins in the uterus might regulate myometrial activity during pregnancy and labor (Burdet et al., 2010).

**FIGURE 7:**
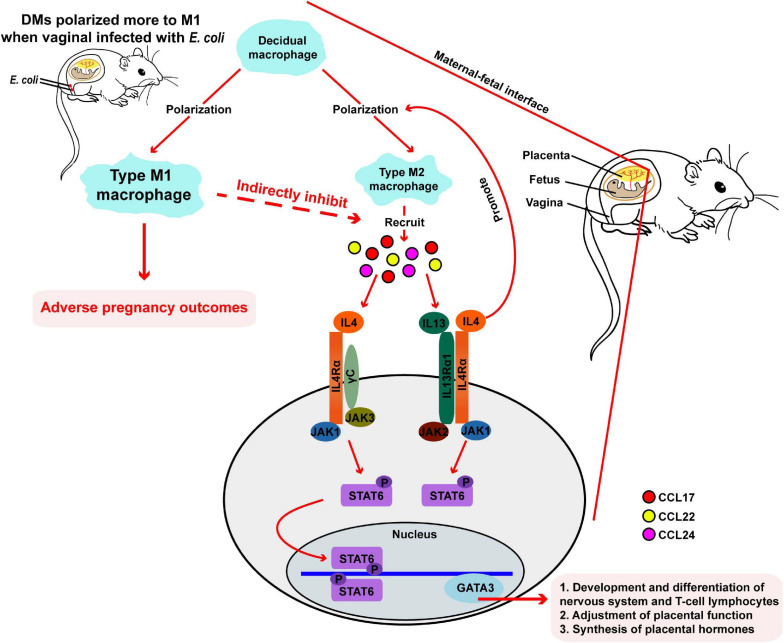
Responses in the maternal-fetal interface during pregnancy in mice. During normal pregnancy, DMs polarized more to type M2 macrophages, following by recruiting some chemokines. Then, gene expression of downstream IL-4, JAK-1, STAT-6, and GATA-3 are upregulated, and pregnancy progresses toward normal. When mice are vaginally infected with *E. coli*, DMs transform more to M1 macrophages and adverse pregnancy outcomes followed.

We developed a mouse model of vaginal infection induced by *E. coli* to denote AV after pregnancy. We found that vaginal infection with *E. coli* in pregnancy could cause adverse pregnancy outcomes, such as weight loss in the mother and fetus. Subsequent mechanistic research revealed that macrophages located at the maternal-fetal interface might have a crucial effect and further affect pregnancy outcomes though the IL-4/JAK-1/STAT-6 signaling pathway in pregnant mice inoculated vaginally by *E. coli* (Figure 1A and Figure 7).

IL-4 is a canonical Th2 cytokine and mainly originates from Th2 cells (Maier et al., 2012). The reported effects of IL-4 include promotion of the maturation of dendritic cells, strengthening anti-coccidia immune responses, and enhancing the oxidative burst response (Wang and Secombes, 2015). IL-4 can be tested during all stages of pregnancy at the maternal-fetal interface and plays a protective role which involves improving vascular function, reducing inflammatory agents, and is a powerful function in successful pregnancies (Chatterjee et al., 2014; Cottrell et al., 2019). Therefore, the production of IL-4 raises during the entire duration of gestation. Correspondingly, our data demonstrated a decreased expression of IL-4 in pregnant mice infected by *E. coli* compared with that in the control group. To further discuss the underlying applications of IL-4, we added moderate amounts of IL-4 to the DMs, obtained from the mouse maternal-fetal interface and documented the conversion from M1 macrophages to M2 macrophages gradually after IL-4 addition, followed by increased expression of downstream genes. IL-4 could therefore be a candidate for the treatment of vaginal infections induced by *E. coli* during pregnancy. Of course, a therapeutic trend in cell experimentation is far from enough, but this still gives us confidence to explore the methods of management. The next reasonable animal for study, as well as large clinical research studies should be designed and carried out in the near future. Additionally, pregnancy, a complicated process, is driven and affected by various factors (Bonney, 2001). Menon and colleagues review that activation of p38 mitogen activated protein kinase (MAPK) in preterm birth is considered to be a pivotal factor in adverse pregnancies (Menon and Papaconstantinou, 2016). Liong and colleagues expound that suppression of overexpressed Kirsten rat sarcoma 2 viral oncogene homolog (K-Ras) in the placenta helps prevent adverse fetal outcomes (Liong et al., 2018). Zhou and colleagues report that phosphorylated protein kinase B (p-AKT) is capable of anti-inflammatory property and that upregulation of p-AKT expression could improve adverse pregnancy outcomes (Zhou et al., 2017). All of these articles reveal the complexity of pregnancy and show that management of adverse pregnancy outcomes should be considered comprehensively from multiple perspectives.

Decidual macrophages account for 20% of total leukocytes, are the second largest leukocyte population, and are a prominent subset of antigen presenting cells during pregnancy (Erlebacher, 2013; Tang et al., 2015). In a normal pregnancy, DMs show an M2-like phenotype that has anti-inflammatory roles (Sica and Mantovani, 2012; Erlebacher, 2013; Ander et al., 2019). However, there is accumulating research regarding the pregnancy process as an inflammatory response (Romero et al., 2007). In this current study, we isolated DMs from the maternal-fetal interface of pregnant mice and identified and classified them by flow cytometry. The more polarized M1 phenotype was observed in the *E. coli* group compared with that in the control group. This observation indicates that more macrophages differentiated into the M1 subtype in pregnant mice suffering from a vaginal infection induced by *E. coli*, and data that are in accordance with precedent studies that reported infections during pregnancy (Li et al., 2017; Zhang et al., 2019). However, an increased number of M1 phenotype macrophages, which are induced by various pathogenic factors such as tumor necrosis factor, interferon-γ, and pathogen infection (Jena et al., 2019), have been associated with some abnormal pregnancy complications (Zhang et al., 2019). PCR results from uterus tissues and placenta tissues exhibited few macrophages of the M2 phenotype in the vaginal tracts of mice in the study group compared with those of the control group, followed by corresponding chemokines covering CCL-17, CCL-22, and CCL-24 that were reduced. The downstream gene expression of IL-4, JAK-1, STAT-6, and GATA-3 then all decreased significantly at different levels.

Other signal molecules that merit attention in this work are STAT-6 and GATA-3 and its potential effects have been expounded. IL-4 binds to its receptor (IL-4Rα) and JAK-1 expression thus increases, which is followed by upregulation of the expression of p-STAT6. p-STAT6 then upregulates expression of GATA-3, and IL-4 is activated again (Yue et al., 2018). STAT-6 is important for the formation and maintenance of maternal-fetal tolerance, as well as enhancement of the immune response (Zhao et al., 2009; Meng et al., 2017). GATA-3 plays a crucial role in the development and differentiation of the nervous system and T-cell lymphocytes, adjustment of placental function, and synthesis of placental hormones (Mirkovic et al., 2015). The reduction of expression of STAT-6 and GATA-3 caused by a decrease in IL-4 expression may be responsible for adverse maternal and fetus outcomes. In this study, we found that expression of STAT-6 and GATA-3 in the uterus and placenta was reduced in the *E. coli* group. Associated with the lower level of IL-4 expression, we considered that these imbalanced molecules might lead to subsequent adverse pregnancy outcomes in our animal model. Of course, more signal pathways need to be elaborated in the future.

Different gestational periods perform different functions. In mice, the presence of a vaginal plug represents formation of fertilized ovum and is usually set as E0.5. E4.5 represents the beginning of embryonic development at the late-blastocyst stage and also the initiation of blastocyst implantation, while E8.5 represents the beginning of organogenesis and the development of extracellular and amniotic cavities (Yockey et al., 2016). Moreover, E11.5 reflects the late stage of limb development, whereas E18.5 denotes fetus delivery. We selected vaginal infection by *E. coli* at E4.5 for investigation and found that adverse pregnancy outcomes (e.g., miscarriage) appeared from E11.5. Further investigation indicated changes at the maternal-fetal interface, and that adverse pregnancy outcomes might be caused by these changes which were expected to be the target, with the purpose of improving the pregnancy outcomes.

The placenta is involved in the transport of nutrients and gasses to the fetus, and removal of waste products from the fetus (Maltepe and Fisher, 2015; Sacerdoti et al., 2018). The usual opinion is that the placenta is a sterile environment, as evidenced by a series of experiments even in abnormal pregnancies (Leiby et al., 2018; Segata, 2019; Theis et al., 2019; Kuperman et al., 2020). However, some studies have shown that there are multiple types of bacteria present in the placenta (Aagaard et al., 2014; Doyle et al., 2014). This disagreement aroused our interest, and we discussed the controversy in our exploration. Three pairs of specific primers for bacteria were designed and agarose gel electrophoresis was performed. The results demonstrated that no bacteria were found in the placenta and even the vaginal infections remained severe. This manifested that the poor pregnancy outcomes we discovered may not have resulted from ascending infections directly. Furthermore, pregnant mice may have been able to protect their fetus from injury by activating some pathways which have not yet been recognized. Pioneering literatures have explored this question – Vornhagen and colleagues supposed that uterine inflammation caused by vaginal infections could eliminate ascending bacteria so as to not induce premature birth (Vornhagen et al., 2016). This view was unsuited to traditional ideas but coincided with our thought, which was that the mother protects their fetus in one way or another. Another article summarized several mechanisms in which the placenta can prevent infections upon exposure to pathogens: a distinct defensive layer, secretion of antitoxic molecules, transport of protective antibodies, and innate immunity barrier (Ander et al., 2019). Of course, more detailed defensive mechanisms still need to be investigated. With respect to the placenta, we found that placental weight at E18.5 was significantly lower in the control group than that in the *E. coli* group. Placental weight has been related to adult cardiovascular disease, diabetes, stroke, and even lifespan (De Paepe et al., 2015). Usually, the higher the placental weight or ratio of placenta-fetus weight, the greater the risk of disease. Similarly, our results support this view. We speculate that pregnant mice might fight against inflammation using various stress responses. After infection and becoming slightly swollen other types of placental weight gain reactions invisible to the eye may take place in the placenta.

Although we have acquired meaningful data in the present study, limitations were inevitable. First, we used *E. coli*-infected pregnant mice for our experiments. Nevertheless, pathogens differ, induced infection vary, and results may change. Therefore, our consequences perhaps only demonstrate the effects of vaginal infection induced by *E. coli* through vaginal injection on the maternal and fetal mouse but may not be suitable for other types of infections or pathogens. However, our work provides a feasible method for investigating the mechanism of certain diseases, particularly for infections during pregnancy. Our research methods of bacteria, referring to *E. coli*, also show the way for detecting the impact of different pathogens on the host. Second, we only infected mice once during pregnancy. Recurrent infections are common in clinical work, but deep research was not possible in this study. Therefore, a comparable study, including infection frequences, infection time, diagnostic methods, and other detailed questions for recurrent infections is essential.

## Conclusion

In conclusion, we established a model of gestational mice with AV infected by *E. coli* and found that vaginal infection induced by *E. coli* during pregnancy in mice could lead to: (i) weight reduction of the mother and fetus and adverse pregnancy outcomes; (ii) reduction in expression of the chemokines, such as CCL-17, CCL-22, and CCL-24 by transformation of M1 macrophages to M2 macrophages; and (iii) a significant decrease in expression of the IL-4/JAK-1/STAT-6 signaling pathway and the downstream gene of GATA-3. Moreover, the placenta was found to be sterile in this trial. Our study offers a potential mechanism for future research of AV management during pregnancy. More detailed research needs to be performed to promote the development of treatment of vaginal infection.

## Data Availability Statement

The original contributions presented in the study are included in the article/Supplementary Material, further inquiries can be directed to the corresponding authors.

## Ethics Statement

The animal study was reviewed and approved by the Animal Research Ethics Board of the Nanjing Medical University.

## Author Contributions

CF, CR, and YD performed the animal trials. CF, CR, LZ, and YF completed statistical analyses. CF, LZ, XW, and TL wrote the manuscript. ZD and WH helped complete the experiments together. PL, QL, and XZ conceived and designed the study, and also offered funding support. All authors contributed to the article and approved the submitted version.

## Conflict of Interest

The authors declare that the research was conducted in the absence of any commercial or financial relationships that could be construed as a potential conflict of interest.

## Footnotes

1 https://geneglobe.qiagen.com/cn/analyze/
