# Supplementary material for: Aerobic Vaginitis Induced by Escherichia coli Infection During Pregnancy Can Result in Adverse Pregnancy Outcomes Through the IL-4/JAK-1/STAT-6 Pathway
Source: Front Microbiol. 2021 Apr 7;12:651426. doi: 10.3389/fmicb.2021.651426 (PMC8058192; doi:10.3389/fmicb.2021.651426)
Supplement: Supplementary file 1 [file Data_Sheet_1.docx]

**Supplementary Table 1: Primer sets.**

| Oligonucleotide | Oligonucleotide PCR primers | Size of Fragment Sequence (bp) |
| --- | --- | --- |
| CCL17 | F: TACCATGAGGTCACTTCAGATGC  R: GCACTCTCGGCCTACATTGG | 103 |
| CCL22 | F: AGGTCCCTATGGTGCCAATGT  R: CGGCAGGATTTTGAGGTCCA | 111 |
| CCL24 | F: TCTTGCTGCACGTCCTTTATT  R: GCATCCAGTTTTTGTATGTGCC | 179 |
| IL4 | F: GGTCTCAACCCCCAGCTAGT  R: GCCGATGATCTCTCTCAAGTGAT | 102 |
| JAK1 | F: CTCTCTGTCACAACCTCTTCGC  R: TTGGTAAAGTAGAACCTCATGCG | 130 |
| STAT6 | F: AGTTCCTGGTCGGTTCAGATGCT  R: TCTCCAAGGTGCTGATGTGCG | 138 |
| GATA3 | F: CTCGGCCATTCGTACATGGAA  R: GGATACCTCTGCACCGTAGC | 134 |
| GAPDH | F: AAGAAGGTGGTGAAGCAGG  R: GAAGGTGGAAGAGTGGGAGT | 111 |
| Bacterial 16S rRNA Primer1 | F: AACMGGATTAGATACCCKG  R: AGGGTTGCGCTCGTTG | 316 |
| Bacterial 16S rRNA Primer2 | F: AYTGGGYDTAAAGVG  R: TACNVGGGTATCTAATCC | 239 |
| Bacterial 16S rRNA Primer3 | F: CCTAYGGGRBGCASCAG  R: GGACTACNNGGGTATCTAAT | 465 |

Note: F: Forward; R: Reverse

**Supplementary Table 2: The results of the fold change screened by the PCR Array.**

| Gene symbol | Fold regulation |
| --- | --- |
|  | **Upregulated** |
| HC | 2.31 |
| IL1b | 2.25 |
| IL24 | 2.11 |
| Cxcl3 | 2.11 |
| Csf2 | 2.08 |
|  | **Downregulated** |
| Cxcl5 | -3.77 |
| Tnfsf11 | -3.28 |
| IL7 | -2.62 |
| IL12a | -2.60 |
| XCL1 | -2.58 |
| IL17a | -2.58 |
| IL21 | -2.58 |
| IL22 | -2.58 |
| IL3 | -2.58 |
| LTA | -2.47 |
| CCL7 | -2.41 |
| IL4 | -2.29 |
| IL13 | -2.29 |
| BMP2 | -2.11 |
| Tnfsf10 | -2.01 |

Note: The expression of MSTN downregulated 4816024.81 folds were not included.

**A**


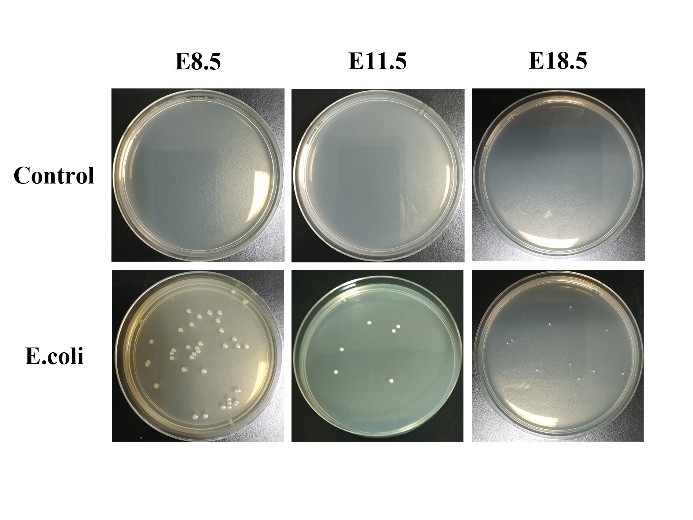


**B**


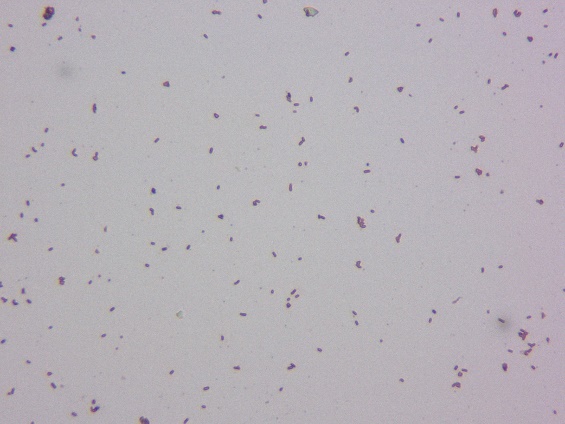


**Supplementary Figure 1** The construction of vaginal infection of mouse model.

**(A)** Cultivating of vaginal lavage fluid in *E. coli* and control group at E8.5, E11.5, and E18.5.

**(B)** Gram staining of vaginal lavage fluid culturing in *E. coli* group.


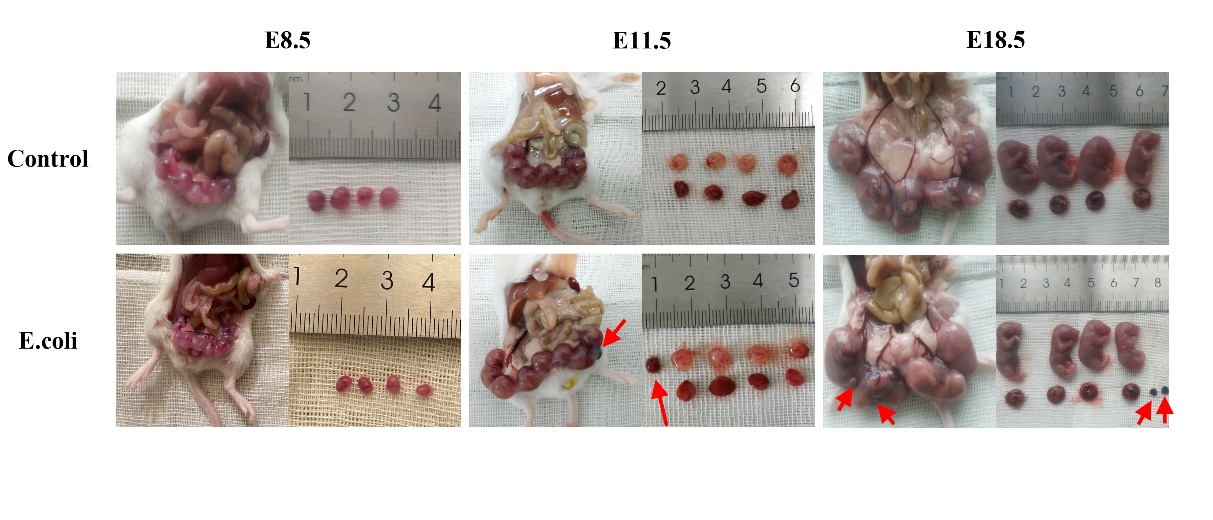


**Supplementary Figure 2** Pictures of cesarean section of fetal mice and placentas in *E. coli* and control group at E8.5, E11.5, and E18.5. The red arrows represent abortive tissues observed at cesarean section.


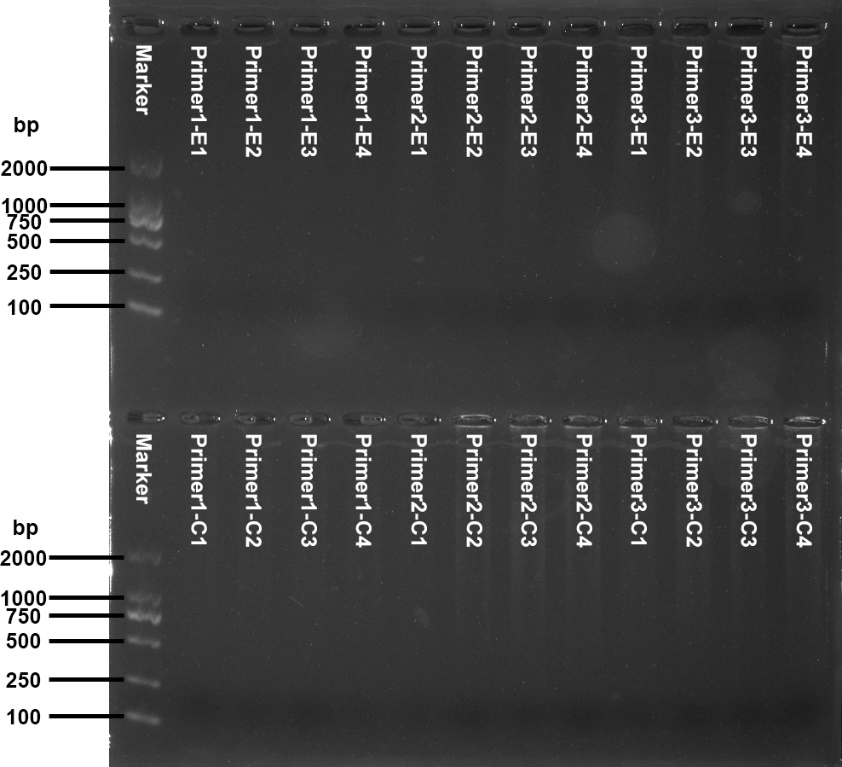


**Supplementary Figure 3** Results of gel electrophoresis of microbial genomic DNA from placenta. Primer 1 to 3 means three pairs of primers designed specifically for bacteria. E1 to E4 represents different samples for *E. coli* group, while C1 to C4 for control group. Marker of gel electrophoresis is on the left.
